# Unpacking the aggregation-oligomerization-fibrillization process of naturally-occurring hIAPP amyloid oligomers isolated directly from sera of children with obesity or diabetes mellitus

**DOI:** 10.1038/s41598-019-54570-8

**Published:** 2019-12-05

**Authors:** Myriam M. Altamirano-Bustamante, Nelly F. Altamirano-Bustamante, Mateo Larralde-Laborde, Reyna Lara-Martínez, Edgar Leyva-García, Eulalia Garrido-Magaña, Gerardo Rojas, Luis Felipe Jiménez-García, Cristina Revilla-Monsalve, Perla Altamirano, Raúl Calzada-León

**Affiliations:** 1 Unidad de Investigación en Enfermedades Metabólicas, Centro Médico Nacional Siglo XXI, Instituto Mexicano del Seguro Social, Mexico city, Mexico; 2 Instituto Nacional de Pediatría, Mexico city, Mexico; 3 Facultad de Ciencias, UNAM, Mexico city, Mexico; 4 UMAE Hospital de Pediatría, Centro Médico Nacional Siglo XXI, Instituto Mexicano del Seguro Social, Mexico city, Mexico

**Keywords:** Protein aggregation, Type 2 diabetes

## Abstract

The formation of amyloid oligomers and fibrils of the human islet amyloid polypeptide (hIAPP) has been linked with β- cell failure and death which causes the onset, progression, and comorbidities of diabetes. We begin to unpack the aggregation-oligomerization-fibrillization process of these oligomers taken from sera of pediatric patients. The naturally occurring or real hIAPP (not synthetic) amyloid oligomers (RIAO) were successfully isolated, we demonstrated the presence of homo (dodecamers, hexamers, and trimers) and hetero-RIAO, as well as several biophysical characterizations which allow us to learn from the real phenomenon taking place. We found that the aggregation/oligomerization process is active in the sera and showed that it happens very fast. The RIAO can form fibers and react with anti-hIAPP and anti-amyloid oligomers antibodies. Our results opens the epistemic horizon and reveal real differences between the four groups (Controls vs obesity, T1DM or T2DM) accelerating the process of understanding and discovering novel and more efficient prevention, diagnostic, transmission and therapeutic pathways.

## Introduction

Any event in the universe of protein conformational diseases (PCD) such as Diabetes Mellitus (DM), Alzheimer Disease (AD), and Cancer, among others, is defined by its information (genetics, epigenetics, molecular networks, stress, ageing, etc) which includes the development of micro and macro-environments and network spheres that influence and interact with each other^1–4^. Proteins are very versatile and fascinating macromolecules; simultaneously the workhorse of the cells and the cornerstone of PCD^5–11^.

All proteins self-organize into their specific conformation in a space of enormous possibilities; a protein with around 100 amino acids can adopt ∼10^46^ conformations and a unique native state which is only marginally stable under physiological conditions. The folding process is error prone and results in the coexistence of misfolded states and off-pathway aggregates^2,12,13^. Therefore, the importance of the protein homeostasis (proteostasis) is demonstrated by the proteostasis network, the strict and energy-dependent mechanisms the cell has in order to prevent protein aggregation which include molecular chaperones and their regulators, the UBPS system, and the autophagy that constitute the proteolytic degradation machineries^1,2,4^.

Human islet amyloid polypeptide (hIAPP) also known as amylin, is an intrinsically disordered protein of 37 residues which is co-expressed and co-secreted with the insulin in the β-cell of the pancreas^14–18^. The hIAPP has a variety of conformational states prone to aggregation through the N-terminus and the central regions^19–25^. Recent studies indicate that aggregation of human islet amyloid polypeptide (hIAPP), especially pre-fibrillar aggregates, or oligomers, is a diabetogenic factor that produces cytotoxicity, progressive β cell failure and death^14,26–40^. We define an oligomer as any number of conformational ensembles of two or more protein molecules in non-native conformation that vary in protein structure, size, number of monomers, cytotoxicity, etc^32,41–44^. Small hIAPP oligomers with a high surface hydrophobicity formed during the nucleation conformational phase are very toxic, permeate the membrane and produce non-selective pores^19,21,26,28,29,32,40,45–47^. These form highly ordered fibrils with cross-β structure although some aggregates are amorphous assemblies^2,12,16,19,48^, the fibers on the surface of the cell membranes are cytotoxic as well by causing the fragmentation of the membrane by micellization^16,40,45,48^; recently was demonstrated that tightly mated β-sheet are relevant for toxicity^24,25,49^. Furthermore, the high resolution structure of hIAPP intermediates in the aggregation pathway was attained by nanodisc stabilization^25^.

The hIAPP expression increases in DM produced proteostasis imbalance by decreasing of proteostasis capacity and increases of hIAPP aggregation and co-aggregation that leads to fragmentation of the mitochondrial network that favor high glycolysis over oxidative phosphorylation, followed by proteostasis collapse countered by the protective metabolism induced by the HIF1α/PFKFB3 metabolic stress pathway that slows β-cell death at the expense of β-cell function^1,2,50–53^. It is well known that aging is the major risk factor for proteostasis collapse but obesity and DM break this paradigm as we show in this work.

Despite decades of works in the field of folding-diseases, open questions remain about the nature of PCD (etiology-phisiopatology-natural history) such as what triggers the proteins aggregations *in vivo* in humans? What is the role of the amyloid oligomers *in vivo* in humans? What do amyloid oligomers share in common? Are they homo or hetero-oligomers? Are the oligomers in the serum as a natural mechanism to clearing? Is there a common mechanism of toxicity?. The answers to these questions have implications in the prevention, diagnosis, treatment, prognosis and transmission of DM and PCD in general.

Our cross-functional project development team of translational medicine accelerates the steps to transfer basic scientific discoveries from laboratory benches to Diabetes clinical application (papers I and II). This is the first report of *Diabetes as conformational disease translational process*, which includes: *Discovery* (paper I): isolation-stabilization, initial ultrastructural morphological-immunoreactivity and biophysical studies on the process of aggregation, oligomerization, and fibrillization of real hIAPP from sera of pediatric patients. We demonstrated that the real hIAPP amyloid oligomers (RIAO) exist in sera as small, medium and large size, they are homo and hetero-oligomers, they form fibers, aggregated very fast and delay the process of fiber formation of synthetic hIAPP. In this way, the value of this study is two-fold, not only is it the first time that real hIAPP oligomers have been used to study their relation to the onset, progression and comorbilities of diabetes and obesity, it opens a new window into the study of amyloid oligomers that may allow further investigation in this field and complements the research using synthetic proteins, this means that the advancement of new diagnostic tools, prevention strategies, and more effective treatments can be furthered by novel insight; changing the physio-pathological paradigms for studying obesity and DM as PCD that are a global health problem with a high economic and social cost^54,55^.

## Results

### Hexamer oligomers as potential biomarkers of early β-cell failure

Biomarker identification of early β-cell failure is multidimensional and encompasses the need for a deep understanding of the aggregation/oligomerization process *in vivo*, their dynamics and their molecular networks^9^. We avoid the polymorphism of oligomeric species by concentrating on those oligomers with low molecular weight that are related to cellular apoptosis and may have a role to play in the developing stages of metabolic syndrome and diabetes^26,31,32,56,57^. We used a potential “universal pre-treatment”; which is cost-effective and easy to implement in diagnostic laboratories in hospitals^34^.

A total of 15 patients from the pilot hIAPP oligomers study were chosen randomly, in the group of Diabetes Mellitus: 5 patients with type 1; 5 with type 2. 5 patients with obesity. All were compared with 5 children from the healthy group (Table 1).

**Table 1:** Clinical characteristics of the participants.

	Control (n = 5)	Obesity (n = 5)	T2DM (n = 5)	T1DM (n = 5)	P
Sex [fem], n (%)	2 (40%)	2 (40%)	3 (60%)	2 (40%)	0.895^†^
Age [months]	166 (133, 183)	159 (124, 188)	174 (129, 202)	158 (133, 210)	0.915^‡^
ZS IMC (DE)	0.21 (−0.05, 0.53)	2.86 (2.63, 3.96)	0.71 (−0.34, 2.55)	−0.46 (−1.07, 1.17)	<0.001^‡^
Evolution [months]	—	—	28 (12, 56)	30 (0, 60)	0.882^‡^
Glucose [mg/dL]	91 (81, 92)	95 (90, 98)	103 (96, 308)	300 (185, 518)	0.013^‡^
HbA1c [%]	5.6 (5.2, 6.0)	4.9 (4.6, 5.1)	7.7 (5.3, 12.8)	12.5 (10.8, 14.0)	0.002^‡^
Toral cholesterol [mg/dL]	92 (74, 140)	151 (105, 184)	169 (100, 312)	178 (123, 212)	0.044^‡^
HDL-C [mg/dL]	43 (33, 57)	33 (31, 40)	50 (32, 68)	41 (38, 48)	0.072^‡^
Triglycerides (mg/dL)	65 (52, 115)	195 (120, 259)	173 (50, 537)	82 (48, 400)	0.066^‡^
Uric acid (mg/dL)	6.4 (3.9, 6.7)	5.5 (3.5, 6.7)	4.3 (3.6, 4.9)	3.6 (3.1, 9.3)	0.463^‡^
Insulin Tx. [Sí], n (%)	—	—	0 (0%)	6 (100%)	0.002^†^
Insulin dosage	—	—	—	0.81 (0.54, 1.14)	—

T2DM: type 2 diabetes mellitus group, T1DM: type 1 diabetes mellitus group, n: sample size. P values were calculated by chi-square teste, if the dependent variable was categorical, and by Welch’s test, if the dependent variable was continuous. The pairs do not share the same letter were different al the level of p < 0.05.

The distribution of clinical characteristics (sex, age, evolution), biochemical characteristics (glucose, HbA1c, total cholesterol, HDL-C, uric acid) and treatment were presented by their diagnostics. Quantitative variables were summarized by median and range, and categorical variables were reported with their absolute and relative frequency. Numerical variables were compared by Welch’s test due to the lack of homoscedasticity and incompatibility with the normal distribution assumption^58^. To compare categorical variables Pearson’s chi-squared test was performed^59^.

**Figure 1:**
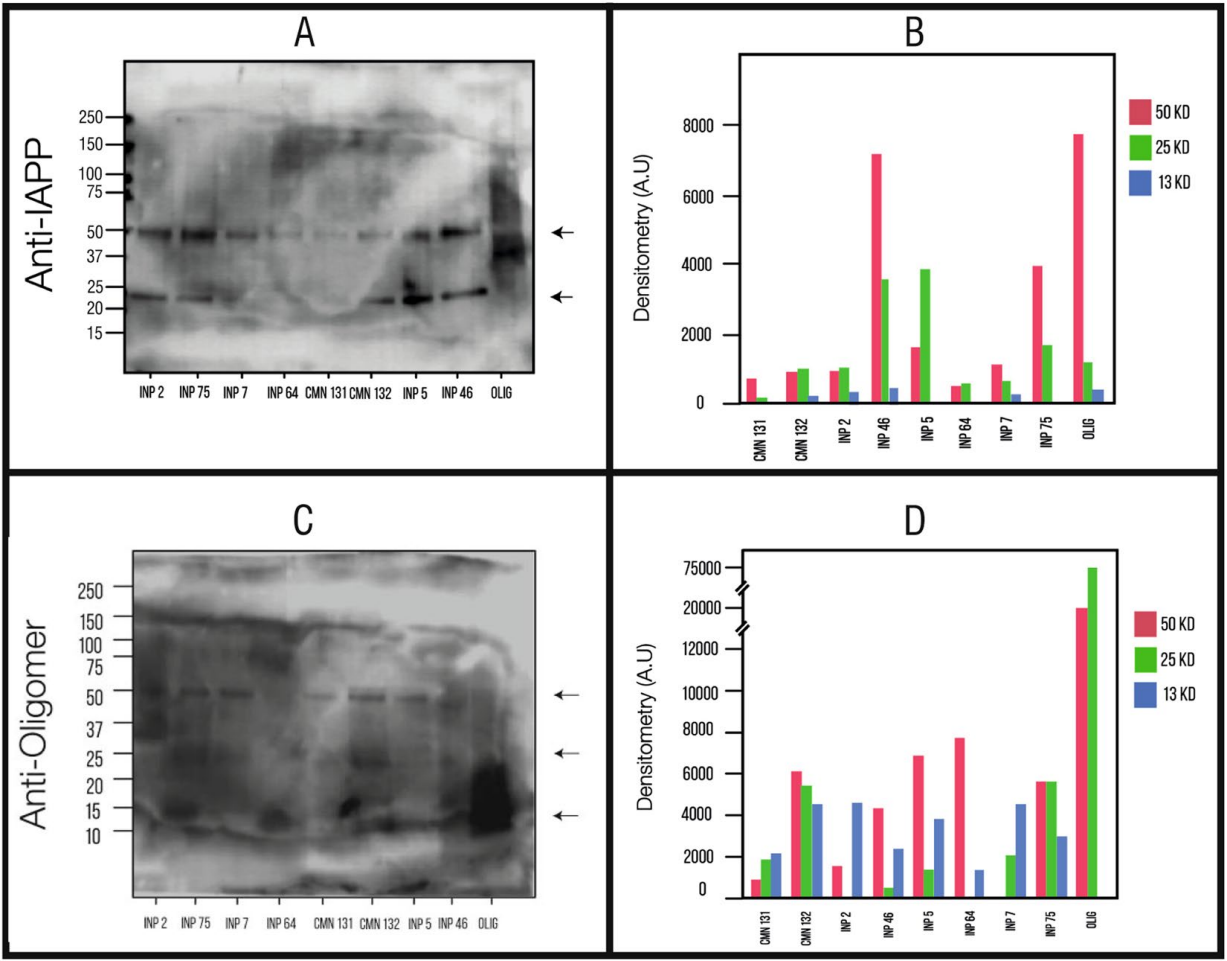
Results from the western blot. (**A**) PTS reacting to the Anti-hIAPP antibody. Codes of samples: (INP2: T1DM, INP75:T2DM, INP7:T1DM, INP64:T2DM, CMN131:Healthy children, CMN132:Healthy children, INP5:T1DM, INP46:T2DM, OLI: hIAPP synthetic oligomers). Ten micrograms of each PTS were loaded in SDS-PAGE 12% gel. The numbers on the left correspond to the molecular weight in kDa for the bands. The largest amount came out in the 50 kDa and 25 kDa markers. (**B**) Densitometry analysis of the samples for the bands of 50, 25, and 13 kDa. It shows us the amount of hIAPP oligomers found in each of these bands for these three molecular weights. (**C**) Samples reacting to the Anti-oligomer antibody. (**D**) Densitometry for the oligomers found in each sample.

We demonstrated for the first time the presence of soluble hIAPP oligomers in pre-treated samples (PTS) of sera of children patients with type 1 diabetes (Group A), type 2 diabetes (Group B) and obesity (Group C) (Fig. 1); we compared the PTS of patients (groups A-C) with the ones of healthy children (Group D) by Western Blotting (WB) using the anti-hIAPP antibody and anti-amyloid oligomer antibody (A11) (Fig. 1). The hIAPP formation of soluble amyloid oligomers is well documented in the literature *in vitro* and *in vivo* in animal models^14,26,41,42,56,60–63^. Recently, Bram and coworkers showed in a small sample of patients that the natural auto-antibodies of patients with diabetes recognized hIAPP synthetic oligomers *in vitro*^34^.

hIAPP oligomers exhibited several aggregation-oligomerization states: Trimers (13 kDa), Hexamers (25 kDa) and Dodecamers (50 kDa) with anti-IAPP antibody (Fig. 1A); hexamers (25 kDa) and Dodecamers (50 kDa) with anti-amyloid oligomer antibody. (Fig. 1C). The hIAPP trimers, hexamers and dodecamers have also been observed in serum of STZ-induced diabetic rats^26^.

Densitometric quantification of each of the oligomerization states labeled with anti-hIAPP and anti-amyloid oligomer showed different oligomerization-profiles. There is significative variation between obesity, type 1 diabetes and type 2 diabetes and the healthy children (Fig. 1B,D). The low molecular weights are predominant and according with the literature where the size of the oligomeric assembly has an inverse correlation with the potency of their toxicity, so we found cytotoxic oligomers (CO) in children with obesity, T1DM and T2DM..

### Wide range of sizes of naturally-occurring hIAPP oligomers and fibers observed in sera of children with obesity or DM

**Figure 2:**
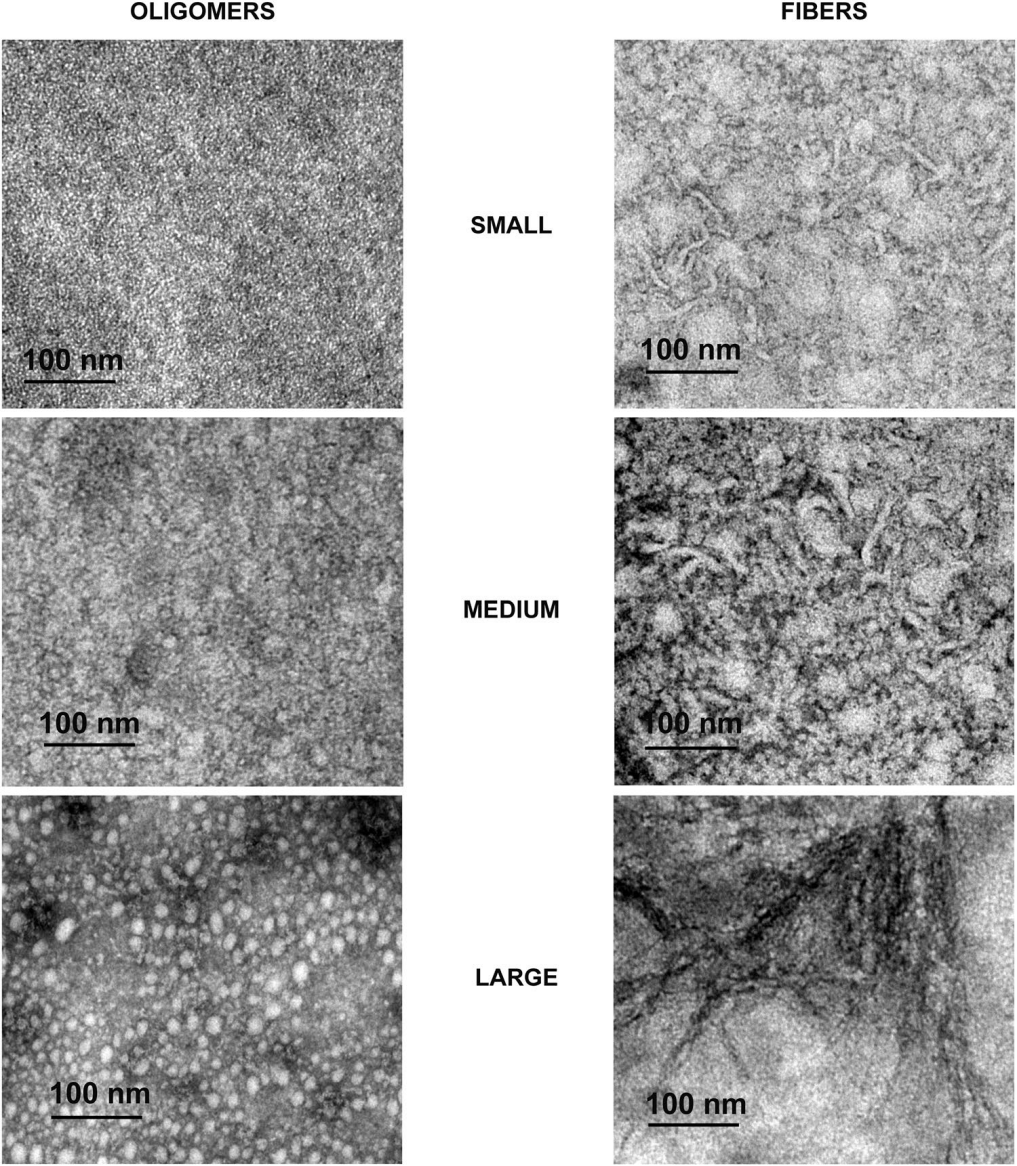
Representative TEM images of Oligomers and fibers characterization in small, medium, and large from the negative staining of patient PTS with different clinical characteristics. PTS at 0.1 mg/mL were in PBS 1X pH 7.4 before TEM experiments.

The morphology of the RIAO from PTS of sera of pediatrics patients from the study population was analyzed by Transmission Electron Microscopy (TEM) (Fig. 2).

**Figure 3:**
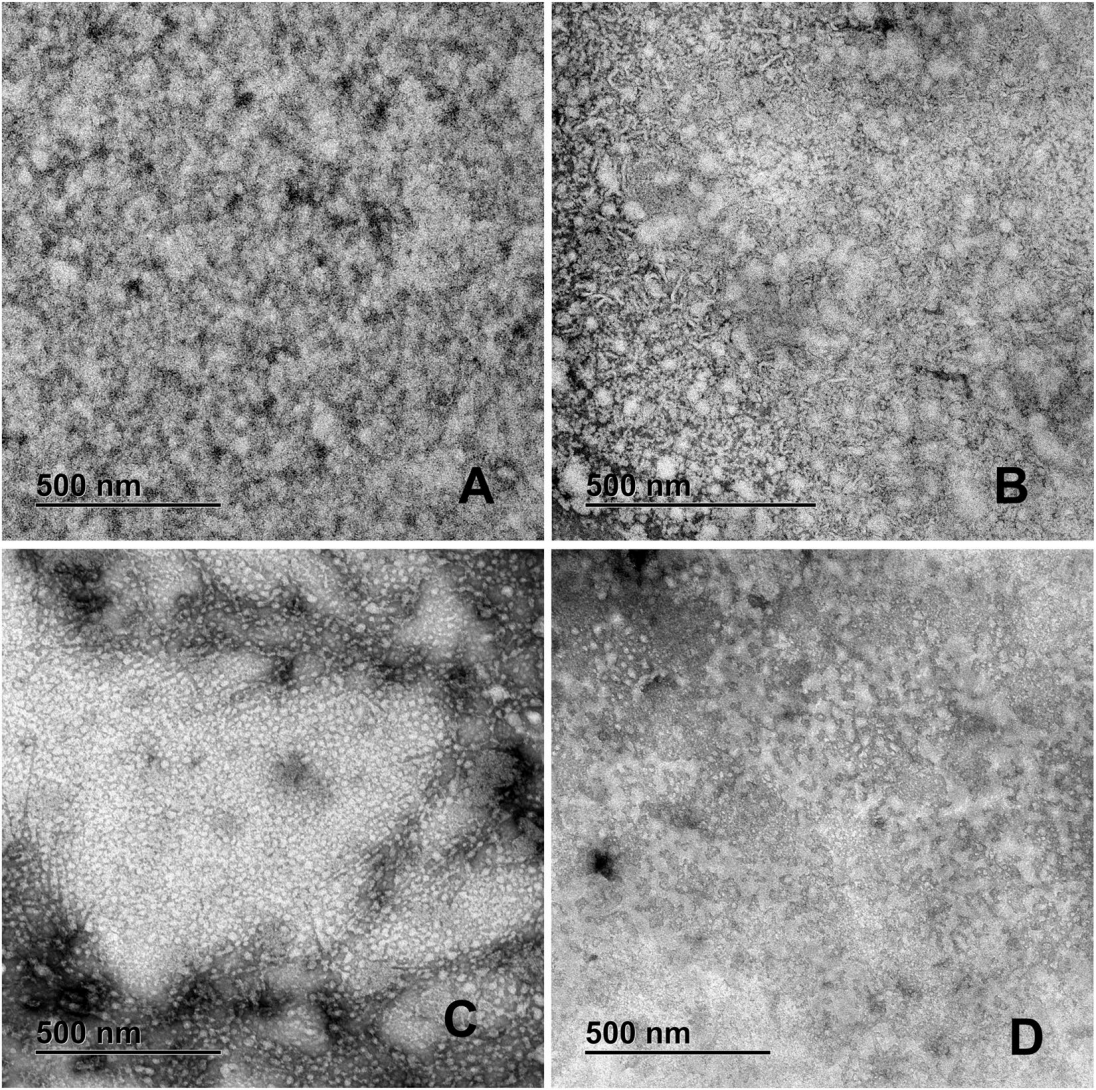
Representative TEM images of Negative staining of the patient PTS. (**A**) Group A-T1DM; many oligomers and a few intermixed fibers. (**B**) Group B-T2DM; We can see large oligomers forming clusters, mixed with small and medium fibers. (**C**) Group C-Obesity; A high amount of medium oligomers is found with very elongated fibers. (**D**) Group D-Healthy children; Small and medium oligomers with small fibers. PTS at 0.1 mg/mL were in PBS 1X pH 7.4 before TEM experiments.

In all the samples the presence of small (~1.05 nm), medium (~16.56 nm) and large (~400 nm) oligomers is evident. In the group A we found a large number of small oligomers, while in the group B the large oligomers predominate; group C had many medium oligomers and group D a large number of small and medium oligomers (Fig. 3).

### The RIAO from PTS from sera of children form amyloid fibers

In many PTS there are observed small (~55.71 nm), medium (~69.21 nm), and large (~252.31 nm) amyloid fibers building a network (Fig. 2). It is a striking result because it revealed the capacity of hIAPP amyloid oligomers to form fibers, and it is an evidence that the aggregation/oligomerization process continues to be active even in the test tube (Fig. 2). The studied groups show: A: small fibres; B: small fibres and medium fibres C: very long fibres located around the oligomers, D small fibres (Fig. 3).

### The RIAO and fibres are recognized by anti-hIAPP and anti-oligomers antibodies in the ultrastructural immunolocalization studies

**Figure 4:**
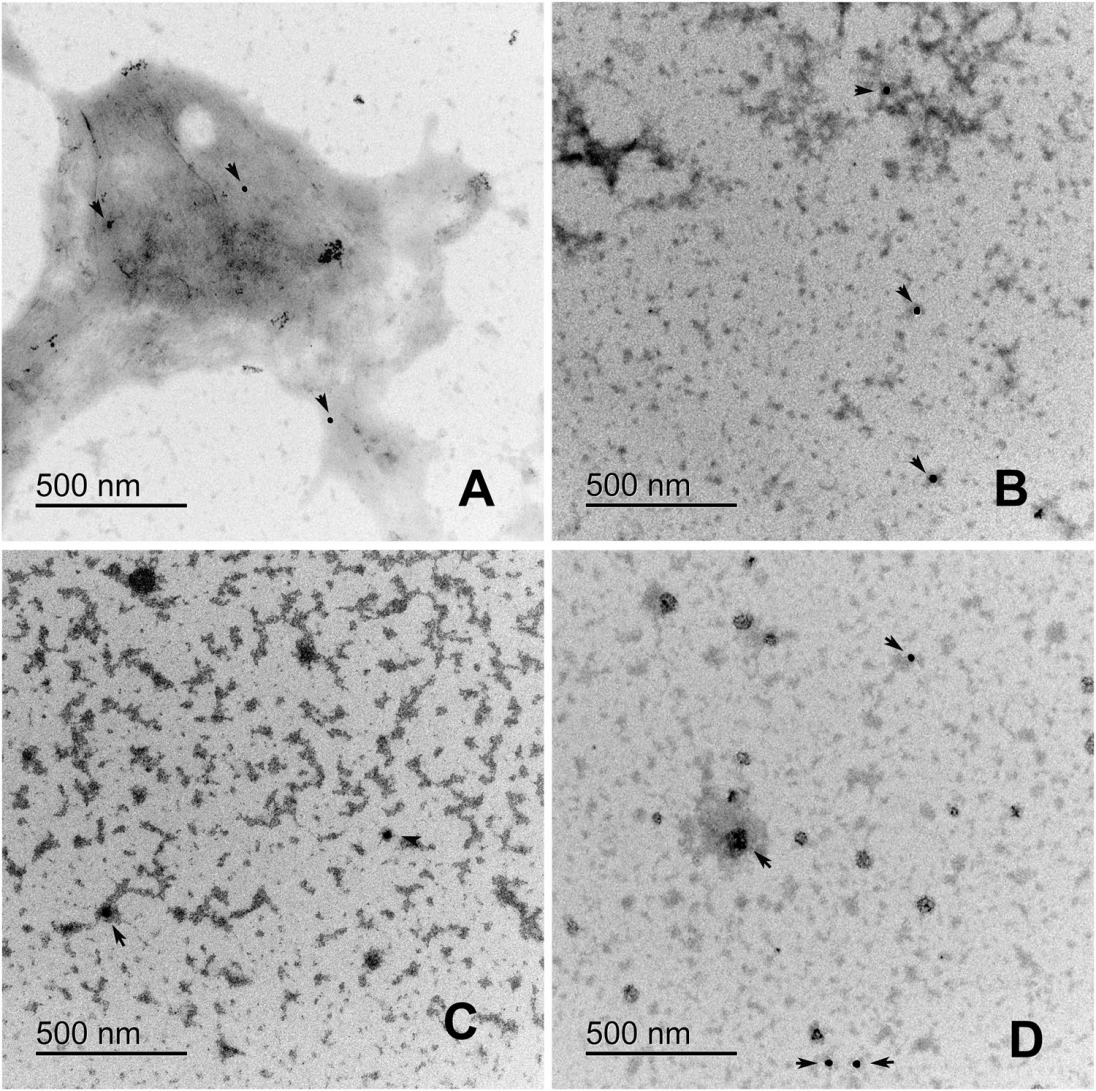
Electron micrography of the immunolocalization of anti-amylin in patient PTS. (**A**) Group A-T1DM, (**B**) Group B-T2DM, (**C**) Group C-Obesity, (**D**) Group D-Healthy children. Colloidal gold is 20 nm. Gold traces are found on small fiber clusters. PTS at 0.1 mg/mL were in PBS 1X pH 74 before TEM experiments.

**Figure 5:**
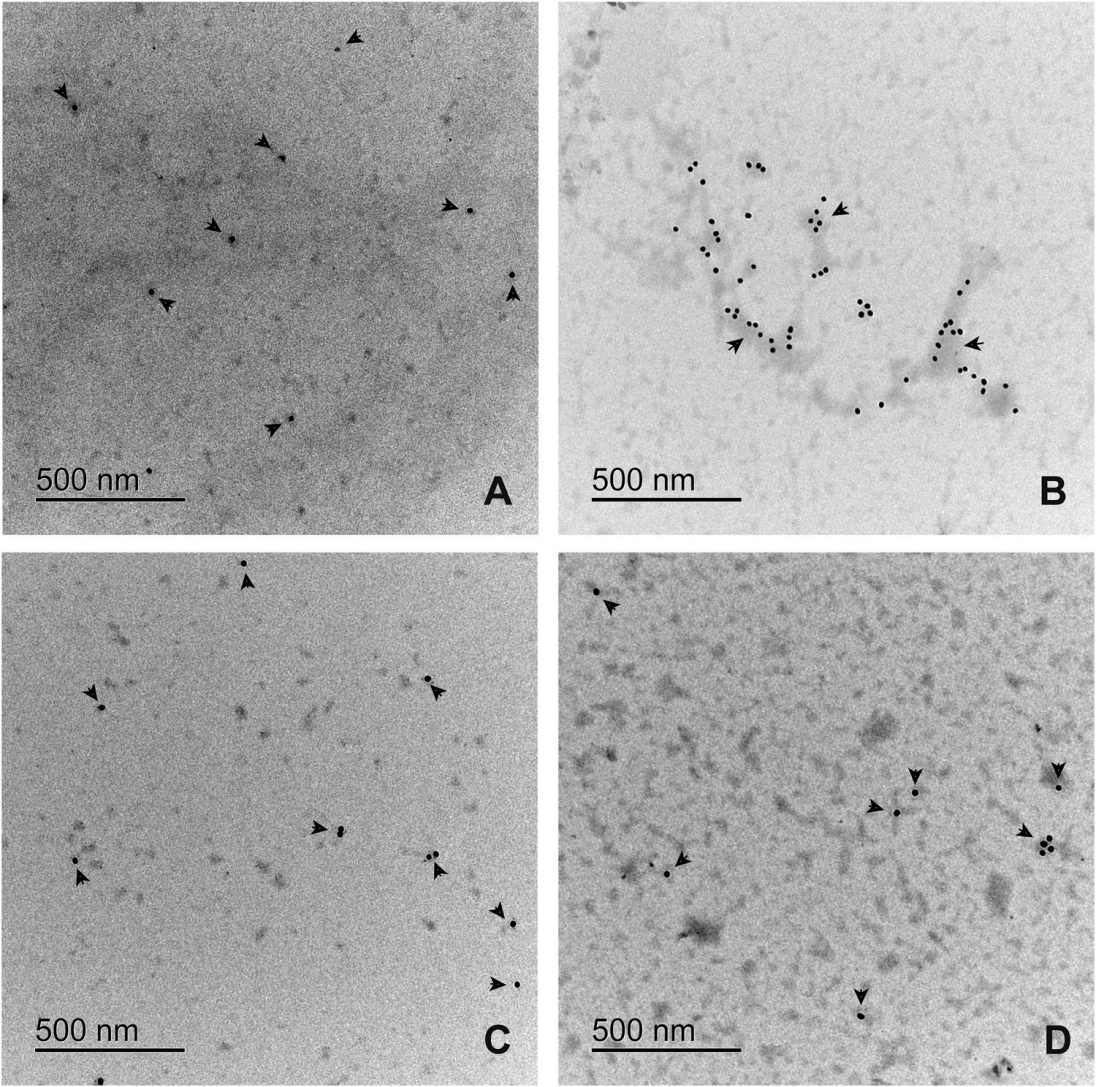
Electron micrography of the immunolocalization of anti-A11 in patient PTS with different clinical characteristics. (**A**) Group A, shows disperse granules (**B**) Group B, we can find the gold particles on the fibers (**C**) Group C and D are similar to figure (**A**). Colloidal gold is 12 nm. Gold traces are found on fibers or fibers clusters. PTS at 0.1 mg/mL were in PBS 1X pH 7.4 before TEM experiments.

Amylin and oligomers antigens recognized by monoclonal antibodies (mAbs), anti-amylin, and A11 (anti-oligomers) were immunolocalized by TEM. Both mAbs were localized on fibres or small clusters of fibres (Figs. 4 and 5) The loss of oligomers is due to the washing process; the fibers adhere better to the formvar membrane. In the immunolocalization of anti A-11 (Fig. 5) we can see a greater amount of gold particle deposits than in Fig. 4. In group A we can see dispersed granules. In group B there are gold clusters on the fibres. Group C and D is very similar to group A, the gold particles are found on small fibre clusters.

### The aggregation of RIAO from samples is fast and delays the process of fiber formation of synthetic hIAPP

**Figure 6:**
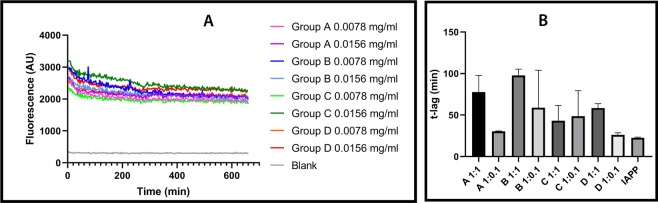
(**A)** Results from the ThT fluorescence experiments. (**A**) Group A-T1DM, (**B**) Group B-T2DM, (**C**) Group C-Obesity, (**D**) Group D-Healthy children. Representative sample from every group was analyzed at two different concentrations, and in absence of synthetic monomeric hIAPP. A blank sample was included for comparison. All measurements were performed with a ThT-buffer at 37 °C (PBS 1X, pH 7.4 + 20 μM ThT); protein concentrations of 0.039 mg/mL or 0.0039 mg/mL (**B**) Comparison of the average t-lag for the trapping experiment (three measurements). One sample from every group was added to synthetic monomeric hIAPP at the same concentration 1:1 (0.039 mg/mL each one), and at one tenth of the concentration 1:0.1 (0.039 mg/mL synthetic hIAPP: 0.0039 mg/mL patient PTS). Synthetic Monomeric hIAPP in absence of patient PTS is included as reference.

We explored the kinetics of aggregation of the RIAO samples, and we found that they aggregated quickly. In Fig. 6A we observed an immediate augmentation in the intensity of the Thioflavin T (ThT) fluorescence that implies that the sample is already aggregated^64,65^. This behaviour is concentration dependent. Then we tested the seeding effects of those oligomers from several samples in the synthetic hIAPP aggregation kinetics (Fig. 6B). We demonstrated that the oligomers from groups A and B delayed the process of fibrillization of the hIAPP (Fig. 6B). They showed a significant increase in the lag phase compared to synthetic hIAPP alone (t-lag 22.8 min to 77.8 min for group A, and t-lag 22.8 min to 97.8 min for group B), thus delaying the toxic effect of the oligomers. Whereas the oligomers from group D showed a smaller delay of the aggregation process (Fig. 6B).

### Exploring the biomolecular assembly of RIAO

We decided to study the hIAPP oligomers from PTS of sera of children with obesity and diabetes from the protein folding perspective. The aggregation/oligomerization/fibril formation process usually implies conformational changes from monomer native structure to the β-sheet-rich structures of amyloid polymorphism. Furthermore, we explored the biomolecular assembly of hIAPP by far-UV Circular Dichroism spectroscopy (CD).

**Figure 7:**
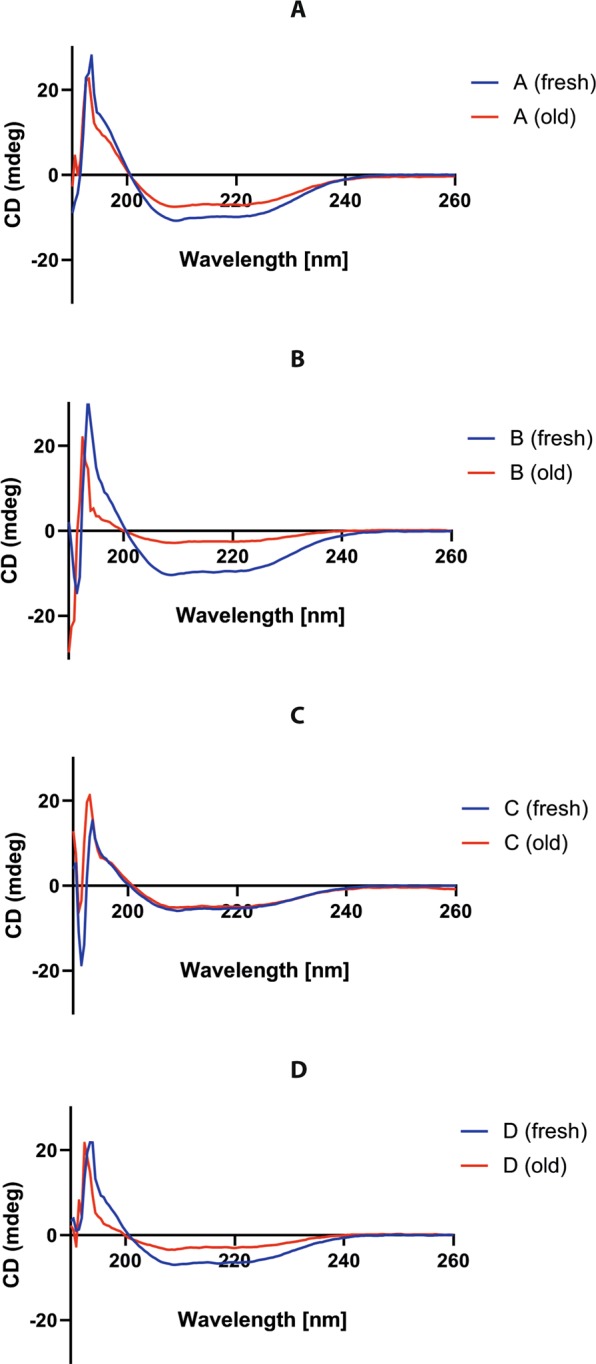
Results for the circular dichroism experiments, comparing the spectra between freshly prepared samples and the same sample after some time in storage. (**A**) Group A-T1DM, (**B**) Group B-T2DM, (**C**) Group C-Obesity, (**D**) Group D-Healthy children. Each letter corresponds to the sample’s group. All measurements were performed with 0.1 mg/mL of patient PTS in PBS 1 X buffer (pH 7.4) and 0.1 cm flow-cell at RT (25 °C).

The CD of freshly and old PTS from each group showed diversity (Fig. 7(A–D)). We followed the development of a β-structure signature and the decrease of α-structure signature. In Fig. 7(A–D) we compare representative samples of each group. We used synthetic hIAPP oligomers as reference molecules. The secondary structure percentages obtained for synthetic hIAPP were 35% turn/sheet, >50% random coil, and 10% helix, which are consistent with the reported in the literature^63,66,67^. The synthetic hIAPP oligomers have >88% turn/sheet, 10% random coil and <8% helical structure^63^.

In the features of CD of secondary structure elements from the samples of all groups A-D (Fig. 7) we found a positive peak at ~190–195 nm and two negative peaks at 208 and 222 nm corresponding a signal of α–helix; also a positive peak ~195–200 nm, and a negative peak ~215–220 nm corresponding with a signal of β–sheet. In general, the CD signal at 216–218 nm indicates the sheet content.

For the representative spectrum of the group A samples in Fig. 7A, we showed that the CD signals at 195 nm, 208 nm, and 222 nm were about 30% higher in the fresh sample. This means that the secondary structure variation on group A samples (expressed in Δ CD signals of old sample – fresh sample in %) is negative for α–helix at 195 nm. The negative signs stand for a decrease in the corresponding structure as described by Juárez. On the other hand, the β–sheet signal at 216 nm increased, which implies that the corresponding structure is increasing. Group B samples display CD signals four times higher at 195 nm, 208 nm, 218 nm and 222 nm in the fresh samples (Fig. 7B). The ΔCD signal is negative at 195 nm and positive at 218 and 222 nm. The positive signs stand for an increase in the β–sheet structure and the unordered structure. Group C samples do not display differences in the CD signals (Fig. 7C), whereas group D showed CD signals around three times higher at 195 nm, 208 nm, 218 nm and 222 nm in the fresh sample (Fig. 7D). The ΔCD signal is positive at 208 nm, 218 nm and 222 nm.

Within the samples from groups A-D analyzed here, the content of α-helical structure is negative and larger for group B, meaning a loss of the structure. The average R1 parameter (ratio 195/208) that characterizes the individual helices is bigger in the old sample of group D, and is ordered: D > A > C > B. The ratio 222/208 nm stands for the presence of single helices, and in all the samples it was about 0.9, indicating a lower inter-helix interaction. Meanwhile, the increase of β–sheet structure and the unordered structure was ordered: B > D > A > C. There was a loss in α-helix structure and formation of both β-sheet and unordered conformation. Regarding the hIAPP homo-oligomers, these findings are well appreciated when we obtained the fraction of secondary structure using the CDSSTR method^68^ (Supplementary Fig. 1).

### The RIAO from sera are homo and hetero-oligomers

**Figure 8:**
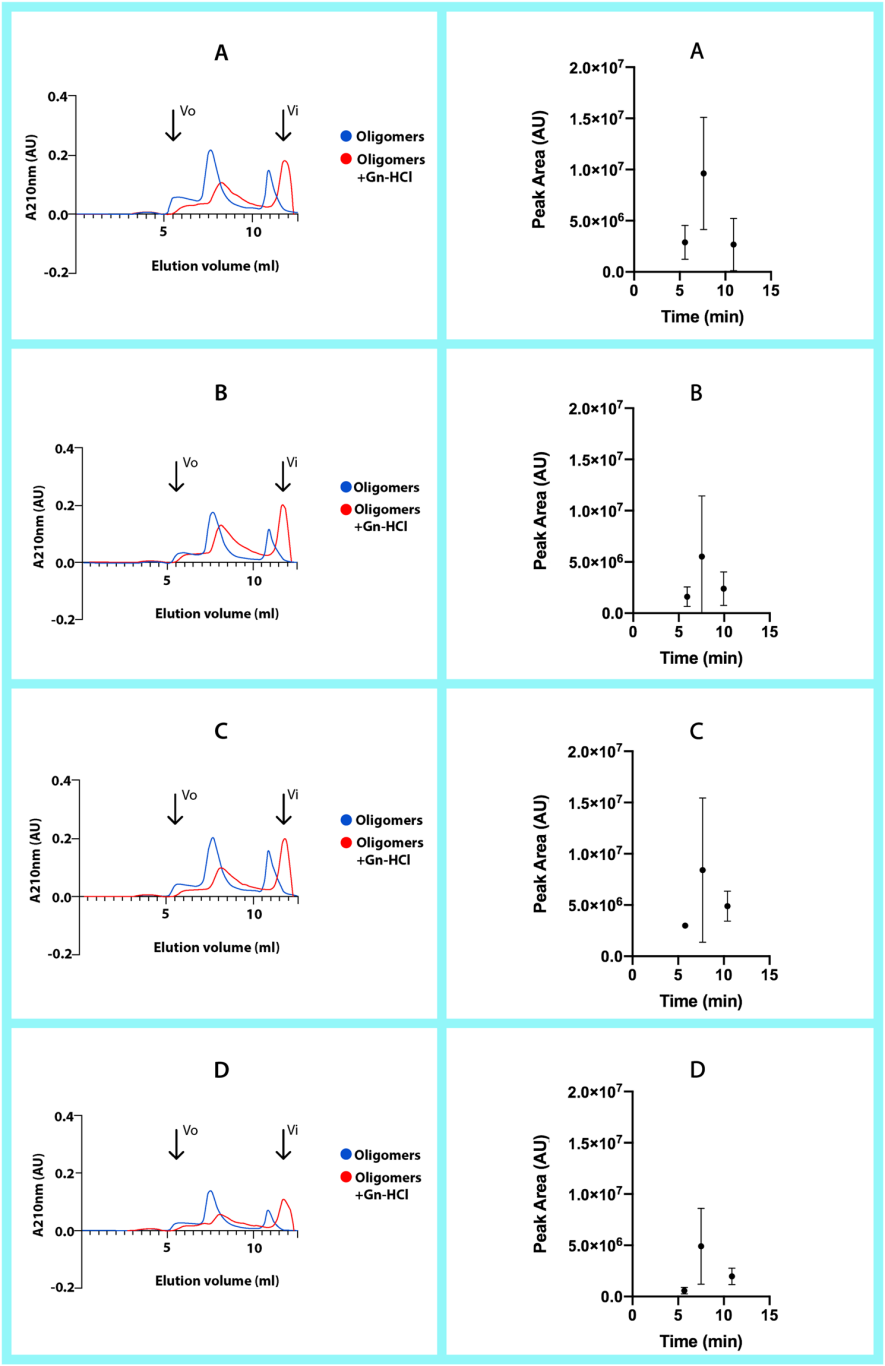
Results from the SEC fractionation experiments on a BioSuite 250, 5 μm HR SEC column. The panels on the left show the chromatography for selected representative PTS from each group, both with and without Gn-HCl as denaturant. The panels on the right show the results for all the PTS run for each group, where we can compare the differences between each sample’s peak area and elution time (without Gn-HCl). (**A**) Group A-T1DM, (**B**) Group B-T2DM, (**C**) Group C-Obesity, (**D**) Group D-Healthy children. All measurements were performed with 0.8 mg/mL of patient PTS or 0.4 mg/mL of patient PTS:Gn-HCl (3 M), in 50 mM Tris-HCl + 50 mM KCl pH = 7.4 at flow rate of 1 ml/min.

Eleven PTS of sera from patients and controls were analyzed by Size Exclusion Chromatography (SEC)^69^. The samples were in absence and presence of 3 M guanidine hydrochloride (Gn-HCl); insoluble aggregates were removed by centrifugation (Fig. 8(A–D)).

The samples in absence of GuHCl eluted at several peaks, and they were very similar to each other from one sample to another (Elution volume of ~5.8 ml-peak 1; ~7.8 ml-peak 2; and ~10.86 ml-peak 3) in the chromatogram of SEC column (Fig. 8A). The size of the peak is what mostly varies between each sample. This indicates that in some samples there is significantly more of the aggregate species corresponding to that peak than in others. In the first peak (~232 kDa) the maximal area is for the samples from group A, and the minimal area for the samples of group D (Fig. 8). It is important to point out that this peak displays strong tyrosine (Tyr) and tryptophane (Trp) signals in the samples of group A, and fair in the samples of group D and the synthetic oligomers. A striking result is that the third peak (~35 kDa) did not display Trp signal.

We compared each group peaks individually by area and by elution time. The results were as shown in Fig. 8. We can observe on the graphs comparing the different peaks’ area percentage at 210 nm that there are greater differences in area between the groups.

Fractionating the PTS with 3 M Gn-HCl yields few elution peaks. The first peak corresponding to the ~208 kDa aggregates disappears, whereas the other elution peaks (elution volume of 6.5 ml, 8 ml and 11.6 ml) correspond to medium and low molecular weight aggregates. The peak corresponding to ~35 kDa is higher taking into account that the concentration of the sample was half of the concentration in absence of Gn-HCl (Fig. 8). We demonstrated that the hIAPP oligomerization is a process of co-aggregations forming homo and hetero-oligomers, since there are peaks with Trp signal and we consider that the hIAPP’s do not have Trp^63^.

Furthermore, we analyzed a sample of a patient from group A by mass spectroscopy (nano electro-spray). The main co-aggregates are: Serum albumin OS Homo sapiens OX 9606 PE 2 SV 1; IGH protein OS Homo sapiens OX 9606 GN IGH PE 1 SV 1; Immunoglobulin heavy constant gamma 1 Fragment OS Homo sapiens; Uncharacterized protein DKFZp686C11235 OS Homo sapiens and Haptoglobin Fragment OS Homo sapiens OX 9606 GN HP PE 1 SV 1. More studies are required to identify and understand the co-aggregation networks.

### Translational impact

The translational process of this study starts with a changing paradigm of seeing DM as PCD^30,70^. The discovery of the amyloid oligomers in the STZ-induced diabetes in rats allows the identification of rIAPP oligomers as biomarkers^26^. This new knowledge leads to the search for markers in humans to be able to give short-time diagnostic results, which is the way to overcome the biomarker dilemma^9^. It also allows to study for the first time RIAO from sera of pediatric patients with obesity or DM and shift the paradigm that the conformational disease are linked to aging^2,71^. This opens the window to clinical confirmation that the hIAPP oligomers are in the sera and can be isolated, stabilized, and performed the initial biophysical characterization of them; the next step is the assay development to identify them in sera in a patient population. The accompanied paper is the continuation of the translational process.

## Discussion

We begin to unpack the ultrastructural morphology, aggregation/oligomerization process and protein assembly of RIAO between the groups of patients studied. We demonstrated the presence of homo and hetero-RIAOs in the sera of children and adolescents by WB, TEM and biophysical studies (Figs. 1–8). We found that the aggregation/oligomerization process is active in the sera; the main aggregates are dodecamers, hexamers, and trimers in the case of homo-RIAO^26,72^ (Fig. 1), and for the hetero-RIAO we identified by mass spectroscopy the main co-aggregation proteins: serum albumin, immunoglobulins and haptoglobin, which are similar to the findings of amyloid interactome *in vitro* using plasm^73^. This work offered crucial information regarding human homo and hetero-oligomers of hIAPP, they are recognized by the anti-amyloid oligomers antibody (A11) that not bind to native proteins, monomers or mature amyloid fibers^41,74^. Furthermore, they are small oligomers (trimers and hexamers as shown in Fig. 1) that several research groups demonstrated that as the sizes of the oligomeric assembly decreases, its toxicity and deleterious membrane effects increases^28,29,32,48,56,75^. The striking results that the densitometry of WB from the samples of healthy children has less amount of small oligomers confirm this reasoning (Fig. 1B,D). The amyloid oligomers can form fibers and these fibers react with anti-hIAPP and anti-amyloid oligomers antibodies which imply the existence of self-catalysis and the creation of cytotoxic oligomers as consequence of fiber formation (Figs. 1–5).

These studies together with the findings that hIAPP_8-20_ oligomerization starts from hexamers^76,77^, the fact that the hexamers are biomarkers and the co-aggregation of the rIAPP oligomers with several proteins in STZ-induced diabetics rats^26^, the co-aggregation of the hIAPP with their amyloidogenic fragments hIAPP [_19-29 S20G_ and _8-20_]^77,78^, and the identification-structural characterization of the hexamer oligomers required for the protein DN6 oligomerization^44^, represent a breakthrough and open an epistemic horizon to the physio-pathology and treatment of PCD^39,40,64,79–81^.

We tracked the fibrillization process and showed that it happens very fast for the patients’ samples and it is a dynamic process that continues in the test tube (Fig. 6A), and contrary to what was expected, they acted almost like chaperones in the presence of synthetic hIAPP, slowing down the process of aggregation despite being unable to cross-seeding the process^72^ (Fig. 6B). A further and more detailed analysis will be necessary to explain this behavior.

The CD studies demonstrated that the homo and hetero oligomerization-fibrillization is a dynamic process with large structural changes and there are differences in the spectra of the RIAOs from one group to another. And last but not least, from the results of the SEC presented in Fig. 8 we can also obtain some interesting conclusions. The sample from the type 1 diabetes (A) was one of the most uniform in the elution times of their peaks, and these peaks were in general bigger in area than the other groups (A > C > B > D). In the samples from obesity (C) and the diabetes type 2 (B) the elution times of peaks of the different samples are more scattered, which suggests that they have more varied compositions (Fig. 8B–C).

Integrating all results we are able to get a more complete picture of the amyloid formation. The control group D in general has the less oligomers and fibers in the TEM and WB experiments (Figs. 1–5), and the peaks and the areas from the SEC experiments are smaller (Fig. 8). Furthermore, the conformational changes with time are smaller than in the patients’ groups (Fig. 7). Each different experiment is like a blind man feeling different a part of an elephant, telling us important information about different aspects of this complex phenomenon. A homework for all the protein scientists is to discover and identify with which other biomolecules the hIAPP co-aggregates, the co-aggregation network and the co-aggregation pathway.

By using real patients’ samples, we obtain novel and relevant results that complement the research using synthetic proteins^2,12,13,19–21,29,61,76,78,82–84^, which are key to unravelling this elusive oligomerization-fibrillization process *in vivo*, so that we can advance more quickly towards the finding of potential novel diagnostic tools, prevention strategies, and more effective treatments of this fastly-growing and harrowing disease^27,40,64,72,76,79,80,85,86^.

## Experimental Section

### Study design

A cross-sectional, analytical, ambispective, blinded pilot study was carried out.

### Study participants

Children and adolescents were recruited from two main Pediatric Hospitals in Mexico: Instituto Nacional de Pediatría (INP) and UMAE-Hospital de Pediatría, Centro Médico Nacional Siglo XXI, Instituto Mexicano del Seguro Social (IMSS). Informed consent was obtained from all participants. The study was conducted with the approval of the Ethics and Research Committees of IMSS and the INP.

All the subjects underwent a complete physical examination that included weight measurement, waist circumference, and blood pressure according to the established standards. In addition a blood sample (after 10 hours of fasting) was obtained and stored at −80 degrees.

The participants were divided in 4 groups:

Control Group (D): 5 healthy adolescents (without apparent acute or chronic disease).

Obesity (Group C): 5 adolescents with clinical data on insulin resistance (central obesity, acanthosis, hyperkeratosis). With normal glycemia.

Diabetes Mellitus groups: 5 adolescents with type I DM (Group A) and 5 adolescents with type 2 DM (Group B).

### Isolation of amyloid oligomers from sera: “Universal pre-treatment of samples”

1000 µL of each serum sample is mixed with 9000 µL of ice-cold methanol/acetic acid solution (33% methanol,4% acetic acid) for 1.5 h at 4 °C in order to precipitate the oligomers. The oligomers were then pelleted (15 min at 16,200 g), resuspended in 300 µL PBS 1X buffer, as described by Bram and coworkers^34^, or in 0.1% NH_4_OH and stored a 4 °C^42^.

### Western blot

Total protein concentration of oligomers from pre-treated samples (PTS) of the sera of either control or patients was determined by BCA assay using as standard curve BSA and hIAPP^26^. The concentration of the samples was normalized to 10 μg. The membranes were probed overnight at 4 °C with the purified anti-amyloid oligomers antibody ab126892 (Abcam, 1:1,000) or anti-amylin antibody (Santa Cruz Biotechnology, 1:200) diluted in phosphate-buffered saline with Tween-20 (PBS-T). To control the charge, we performed immunoblotting with Anti-Transferrin (Santa Cruz Biotechnology sc-30159 1:500).

The membrane activity was detected by substrate chemiluminescence (Immobilion Chemiluminescence HRP Substrate 1:1) and revealed by Licor C-digit. The intensity of proteins bands was quantified by Image Studio Lite by scanning densitometry. Data was managed with MS Excel, while statistics and graphs were obtained with SAS JMP 9 statistical package.

### Transmission electron microscopy (TEM)

#### Negative staining

The PTS containing the amyloid oligomers were sonicated in ice (5 min) and applied (5 μL) to 300-mesh formvar/copper grids and left for 5 minutes and then the excess solution was removed. Grids were stained with 3% filtered uranyl acetate for 1 min. After air drying, grids were examined with an electron microscope JEM-1010, (JEOL,Japan). The images were taken by a CCD Gatan Orius SC600 and Digital Micrograph software.

#### Ultrastructural immunolocalization

For immunogold labelling 6 μL pre-treated sample were sonicated in ice for 5 minutes and applied onto 200-mesh formvar/gold grids for 10 minutes and then the excess solution was removed with filter paper. Without letting the samples dry, they were floated over the primary corresponding antibody (anti-Amylin or A11) in a PBS 1X solution 1:100 and left all night at 4 °C in a humid chamber. Afterwards, the grids were washed with PBS 1X and floated over the secondary antibody connected to 20 nm gold particles for the antibody against Amylin and 12 nm for the antibody against A11 (Jackson ImmunoResearch) in solution 1:100 for an hour at room temperature in a humid chamber. Then the grids were washed with water by flotation and left to dry to be contrasted with uranyl acetate at 3% for 30 seconds. The grids were observed under a JEM-1010 (JEOL Japan) electronic microscope and the image capture was carried out with a CCD camera model Gatan Orius SC600 and the digital micrograph software.

### CD spectroscopy

CD spectra were obtained by using a Jasco J-815 spectrometer (Tokio, Japan). PTS from controls (4) and patients (12) were analyzed by using PBS buffer as a blank. Five iterations of each spectra were recorded in the range of 190 to 260 nm; data interval of 0.5 nm, with a scanning speed of 50 nm/min, and a bandwidth of 1 nm; with protein concentrations of 0.1 mg/mL in PBS buffer (pH 7.4) and 0.1 cm flow-cell at Room Temperature (25 °C), coupled to the ASU-autosampler accessory. Results were analyzed in DichroWeb to obtain the fraction of secondary structure using CDSSTR and K2D methods^68^.

### Fluorescence

Kinetics of aggregation-oligomerization. The aggregation process was monitored by ThT Fluorescence, Tyr and Trp fluorescence, and turbidity using a M1000 Tecan (Austria). Briefly, the pre-treated sera of patients and healthy controls were added into prewarmed ThT-buffer at 37 °C (PBS, pH 7.4 + 20 μM ThT). For synthetic hIAPP, the 1 mg/ml of peptide was dissolved in DMSO^64^. All of the experiments were performed in triplicate.

### Size-exclusion chromatography (SEC)

SEC was performed using an Alliance- Waters HPLC Chromatography, on BioSuite 250, 5 μm HR SEC column (Waters, USA) at Room Temperature (25 °C) and equilibrated 50 mM Tris-HCl + 50 mM KCl pH = 7.4 at flow rate of 1 ml/min.

### Ethical approval

The study protocol (2010-785-052) was approved by the Ethical Committee of Instituto Mexicano del Seguro Social and the Ethical Committee of Instituto Nacional de Pediatría. The study was performed in accordance with the International Harmonization Conference guidelines on Good Clinical Practice; all methods were performed in accordance with the relevant guidelines and regulations. Prior to participation in the study all of parents or legal guardians of the participants provided written informed consent to participate in the study and granting the authors permission to use and publish the data and results of this study.

## Supplementary information

Supplementary information

## Data Availability

All relevant data are within the paper and its Supporting Information file.
