# Supplementary material for: Unpacking the aggregation-oligomerization-fibrillization process of naturally-occurring hIAPP amyloid oligomers isolated directly from sera of children with obesity or diabetes mellitus
Source: Sci Rep. 2019 Dec 5;9:18465. doi: 10.1038/s41598-019-54570-8 (PMC6895187; doi:10.1038/s41598-019-54570-8)
Supplement: Supplementary file 1 — Supplementary information [file 41598_2019_54570_MOESM1_ESM.docx]

**Supplementary information**

**Unpacking the aggregation-oligomerization-fibrillization process of naturally-occurring hIAPP amyloid oligomers isolated directly from sera of children with obesity or diabetes mellitus.**

Myriam M. Altamirano-Bustamante^1*,&^, Nelly F. Altamirano-Bustamante^2&^, Mateo Larralde-Laborde^1&^, Reyna Lara-Martínez^3^, Edgar Leyva-García^1^, Eulalia Garrido-Magaña^4^, Gerardo Rojas^4^, Luis Felipe Jiménez-García^3^, Cristina Revilla-Monsalve^1^, Perla Altamirano^1^, Raúl Calzada-León^2^

^1^Unidad de Investigación en Enfermedades Metabólicas, Centro Médico Nacional Siglo XXI, Instituto Mexicano del Seguro Social, Mexico city, Mexico.

^2^Instituto Nacional de Pediatría, México city, México.

^3^Facultad de Ciencias, UNAM, México city, Mexico.

^4^UMAE Hospital de Pediatría, Centro Médico Nacional Siglo XXI, Instituto Mexicano del Seguro Social. Mexico city, Mexico.

^&^Contributed equally to this article

*To whom correspondence should be addressed: M.M.A-B ( [myriamab@unam.mx](mailto:myriamab@unam.mx))

**Supplementary figure legends**

**Supplementary Figure 1.** Results for the circular dichroism experiments, comparing the spectra between freshly prepared samples and the same sample after some time in storage. A) Group A-T1DM, B) Group B-T2DM, C) Group C-Obesity, D) Group D-Healthy children. Each letter corresponds to the sample’s group. All measurements were performed with 0.1 mg/mL of patient PTS in PBS 1 X buffer (pH 7.4) and 0.1 cm flow-cell at RT (25 °C).

hIAPP homo-oligomers fraction of secondary structure using the CDSSTR method
